# An Experimental Investigation of R600a Condensation in a Multiport Microchannel

**DOI:** 10.3390/mi15050618

**Published:** 2024-05-01

**Authors:** Burak Çoban, Lütfullah Kuddusi

**Affiliations:** 1 Mechanical Engineering Programme, Graduate School, Istanbul Technical University, 34469 Maslak, Istanbul, Turkey; 2 Mechanical Engineering Department, Faculty of Mechanical Engineering, Istanbul Technical University, 34437 Gumussuyu, Istanbul, Turkey; kuddusi@itu.edu.tr

**Keywords:** microchannel, R600a, condensation, heat transfer coefficient, multiport, refrigeration

## Abstract

This study aims to provide condensation heat transfer coefficients of R600a (isobutane) refrigerant under mass fluxes between 50 and 98 kg/m^2^·s at saturation temperatures of 35 °C, 40 °C and 45 °C. Additionally, experiments are conducted with varying inlet vapour quality to understand its effect on the condensation heat transfer measurement. An aluminium multiport microchannel with a hydraulic diameter ($D_{h}$) of 0.399 mm is used, where a plexiglass cover is mounted on the top of the microchannels to observe the flow conditions. A 1D heat transfer through the aluminium block is assumed, and heat flux through the refrigerant to the coolant is measured to obtain condensation heat transfer coefficients of R600a. The results showed that decreasing saturation temperature and increasing vapour quality increase the condensation heat transfer coefficient. Increasing refrigerant mass flux increases the heat transfer coefficient up to a specific mass flux. It is observed that the effect of inlet vapour quality becomes significant as introduced quality decreases due to increasing fluctuation.

## 1. Introduction

Two-phase flows in microchannels are becoming a very popular topic due to recent advancements in the industry. The footprint of microprocessors is decreasing, yet with increasing computing capabilities, the need arises for higher cooling fluxes. In the refrigeration industry, saving space for food compartments by reducing the size of condensers or evaporators is desired to achieve more compact designs. This higher heat transfer need can only be met by phase-changing flows in microchannel applications, where heat transfer ability is extremely increased compared to macroscale applications. Moreover, using less refrigerant in cooling systems contributes to efforts to decrease global warming.

Condensation occurs when heat is removed from the refrigerant; the gas phase changes into the liquid phase while temperature and pressure remain constant. In two-phase flows, flow regimes play an important role in heat transfer, which refers to the distribution of gas and liquid phases in a channel cross-section. The liquid phase becomes an extra intermediate layer for heat transfer from the gas phase to the ambient, which slows down the heat transfer rate. However, in microscale flows, due to the increasing role of surface tension whilst the gravitational force weakens, the condensed liquid spreads through the channel surfaces. This creates a thinner insulating layer for heat transfer compared to macroscale flows, which, in turn, increases the heat transfer rate.

Various studies observing multi-phase flows in microchannels mainly focus on measuring heat transfer coefficients and pressure losses for different refrigerants. Studies related to condensation are much rarer compared to evaporation. The parameters that authors often investigate are varying refrigerant mass fluxes, hydraulic diameters, cooling heat fluxes and saturation temperatures.

Al-Zaidi et al. [1] investigated the condensation of HFE-7100 experimentally in a rectangular multiport microchannel with a hydraulic diameter ($D_{h}$) of 0.57 mm. The authors stated that increasing the mass flux or vapour quality (x) of the refrigerant increases the local condensation heat transfer coefficient (h) and lengthens the annular flow region. However, coolant seemed to have a negligible impact on the condensation process. The local heat transfer coefficient was unaffected by changing the mass flux or inlet temperature of the coolant, as the authors reported, which changes the wall temperature of the microchannel and the liquid–gas interphase layer temperature. However, the authors also reported that the flow regime at low vapour qualities is shifted to a slug and bubbly flow from annular for all refrigerant mass fluxes when the coolant temperature is decreased.

Rahman et al. [2] investigated the condensation of R134a in rectangular multiport microchannels with ($D_{h}$: 0.64 mm) and without ($D_{h}$: 0.81 mm) fins inside the channels under different saturation temperatures (30 °C and 35 °C) for different mass fluxes (from 50 kg/m^2^·s to 200 kg/m^2^·s). The authors found that a lower saturation temperature has a higher condensation heat transfer coefficient due to the increasing shear stress, surface tension and thermal conductivity of the liquid phase. The authors stated that microchannels with inner fins performed better due to the increased surface area and thinner liquid film thickness. They compared their experimental results with correlations given in the literature and found that their results did not agree well with the literature. Therefore, the authors proposed a new correlation, which has a mean absolute error (MAE) of 17.4%.

Park et al. [3] experimentally investigated the condensation heat transfer of R1234ze (E), R134a and R236fa in a multiport microchannel with a hydraulic diameter of 1.45 mm. The authors reported that the measured heat transfer coefficient did not change by changing inlet vapour quality. On the other hand, increasing the saturation temperature resulted in a decrease in the heat transfer coefficient. The authors compared their data that fell into the annular region to the literature and commented that the agreement was inadequate. Expected outcomes such as increasing vapour quality and mass flux increasing the heat transfer coefficient are also reported in this work.

Singh et al. [4] investigated R134a and R410A condensation in multiport microchannels with hydraulic diameters of 0.666 mm, 0.823 mm and 1 mm. The authors conducted experiments for mass fluxes between 200 kg/m^2^·s and 600 kg/m^2^·s and for saturation temperatures of 30 °C and 40 °C. The authors found that decreasing the saturation temperature increases the heat transfer coefficient and so does increasing mass flux and vapour quality. R134a has higher heat transfer coefficients compared to R410A due to higher surface tension. Additionally, the smallest hydraulic diameter block ($D_{h}$: 0.666 mm) has the highest heat transfer coefficients, as the authors stated. The calculated MAEs varied greatly, from 13% to 317%.

Bohdal et al. [5] experimentally investigated local condensation heat transfer coefficients of refrigerants R134a, R404A and R407C in single microchannels, which have diameters ranging from 0.31 mm to 3.3 mm. They used a compressor in their experimental facility rather than a gear pump, contrary to most researchers. The authors compared their findings to the literature and commented that the accuracy of those correlations is limited to their given specific experimental conditions. Thus, they proposed a new correlation which predicts the experimental data they collected in the range of ±25%. The measured condensation heat transfer coefficient of R134a decreased from nearly 45 kW/m^2^·K to 13 kW/m^2^·K as vapour quality went from 0.35 to 0.025 in the 0.31 mm diameter tube. On the other hand, R404A had less change, from 50 kW/m^2^·K to slightly under 40 kW/m^2^·K, as vapour quality decreased from 0.8 to 0.1. For diameters 1.4 mm, 1.6 mm and 3.3 mm, the authors measured heat transfer coefficients of R134a slightly increased when vapour quality reached 0.7 from 1, which was an unexpected outcome.

Kim and Mudawar [6] investigated the condensation of the FC-72 refrigerant in a 1 mm × 1 mm multiport microchannel module at mass fluxes ranging from 68 to 367 kg/m^2^·s and at saturation temperatures ranging between 57.2 and 62.3 °C under several coolant mass fluxes. Their findings showed that the annular flow region has higher heat transfer coefficients than other regions, and increasing the coolant mass flux decreases the heat transfer coefficient. Authors argue that correlations given for macrochannels had more agreement with their findings rather than those stated for microchannels. The MAE of average heat transfer coefficients ranged from 8.42% to 160%.

Several works related to R600a, such as Lee et al. [7], investigated R600a condensation in a 10.07 mm diameter tube along with R290, R1270 and R22. A double tube configuration is used where water flows in the outer tube for cooling and refrigerant flows in the inner tube with the help of a compressor. The authors showed that heat transfer coefficients decrease with the decrease in vapour quality. Darzi et al. [8] investigated R600a condensation in round ($D_{h}$: 8.7 mm) and flattened tubes ($D_{h}$: 5.1–8.2 mm) for mass fluxes 154.8–265.4 kg/m^2^·s. The authors found that the heat transfer coefficient decreases with decreasing vapour quality and mass flux, and flattened tubes have higher heat transfer coefficients compared to the round tube. Lee and Son [9] investigated R290, R600a, R134a and R22 condensation at 40 °C saturation temperature where pipe diameters ranged from 5.8 mm to 10.07 mm. The authors stated that R600a has the highest heat transfer coefficient amongst other refrigerants, and its heat transfer coefficient increases with decreasing pipe diameter. Basaran et al. [10] numerically investigated heat transfer coefficients for R600a condensation in a 0.4 mm diameter single tube for mass fluxes between 200 and 600 kg/m^2^·s. The authors applied the VOF model with uniform cooling flux. They reported average heat transfer coefficients for different inlet vapour qualities (0.3–0.5–0.7 and 0.9), and their findings show that with increasing mass flux and inlet vapour quality, the heat transfer coefficient increases. Başaran and Yurddaş [11] investigated R600a condensation in a multiport microchannel condenser by applying a thermal simulation model for different pass arrangements and port hydraulic diameters where a Nu number for R600a condensation is used according to their CFD results. Then, an experimental study is carried out to understand the performance of the thermal simulation model for a specific configuration ($D_{h}$: 0.681 mm) by comparing calculated and measured outlet temperatures and pressures of R600a and total heat transfer rates. The microchannel condenser had louvered fins on the air side, and the coolant air temperature was fixed to 22 °C, where the mass flux of R600a was changing between 20 and 60 kg/m^2^·s at the ports. The authors stated that as the hydraulic diameter decreased, the heat transfer coefficient increased; however, the heat transfer capacity decreased.

Pham et al. [12] investigated condensation heat transfer coefficients and pressure drops of R410A, R22, R32 and R290 experimentally in a multiport channel with a hydraulic diameter of 0.83 mm at 48 °C saturation temperature. The authors stated that with increasing vapour quality, the heat transfer coefficient of R290 increases, and at low mass fluxes (such as 50, 100 and 150 kg/m^2^·s), heat transfer coefficients were similar. Amongst refrigerants, R290 was found to have higher heat transfer coefficients, and the authors explained that this result was due to its thermophysical properties. Del Col et al. [13] investigated the pressure drop and condensation heat transfer characteristics of R290 in a 0.96 mm horizontal circular channel at a saturation temperature of 40 °C. The mass fluxes investigated ranged from 100 kg/m^2^·s to 1000 kg/m^2^·s, and the heat transfer coefficients measured increased with increasing mass flux and vapour quality. Del Col et al. [14] investigated the same diameter and saturation temperature with mass fluxes ranging from 80 kg/m^2^·s to 1000 kg/m^2^·s for R1270 condensation. For mass fluxes between 80 kg/m^2^·s and 150 kg/m^2^·s, the heat transfer coefficients found were closer to each other, and the difference becomes less significant as vapour quality decreases.

This study aims to contribute to the literature by providing condensation heat transfer coefficients of R600a for a hydraulic diameter as small as 0.399 mm, which, to the best of the knowledge of the authors, has not been studied before by using the experimental methodology of this work. The data provided in this work can be used to design microchannel condensers for refrigerators or any other thermodynamic refrigeration cycle system with a working fluid of R600a.

Local condensation heat transfer coefficients of R600a were investigated experimentally for low refrigerant mass fluxes (50–98 kg/m^2^·s) in a multiport microchannel. This helps to understand heat transfer behaviour along the condenser pipe or channel at each specific vapour quality rather than using an average value. Additionally, the research is conducted for three different saturation temperatures (35 °C, 40 °C and 45 °C) to investigate the effect of changing the thermophysical properties of R600a on the heat transfer coefficient to comprehend a wider saturation range. Moreover, it was also aimed to understand whether varying vapour quality has an impact on measured heat transfer coefficients when the length of microchannels is not sufficient to observe a full-length condensation. Thus, experiments are conducted with different inlet vapour qualities for each mass flux and saturation temperature. Meanwhile, the coolant had a fixed inlet temperature and flow rate to simulate the actual working conditions of a refrigeration cooling system. It is believed that the present work will enrich the open literature by introducing experimental data regarding the R600a refrigerant.

## 2. Experimental Methods

### 2.1. Experimental Setup

**Figure 1:**
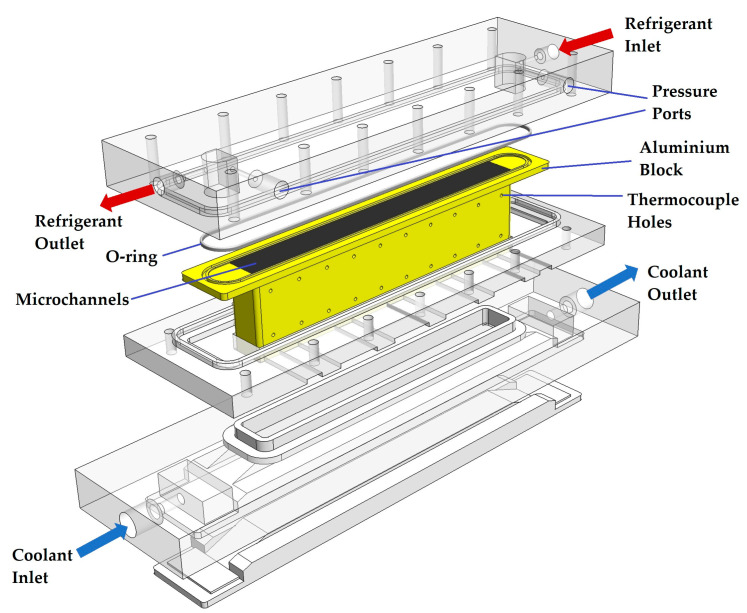
The exploded view of the aluminium block assembly.

Heat flux from the refrigerant side to the coolant water side through an aluminium block is measured to obtain condensation heat transfer coefficients for each vapour quality; a similar method is followed in the works of [1,4,6]. The assembly of the aluminium block is given in Figure 1.

**Figure 2:**
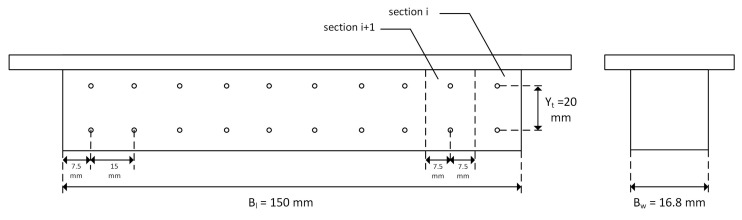
The side and back view of the aluminium block and drilled thermocouple holes.

Twenty measurement holes are drilled into the side of the aluminium block to place T-type thermocouples to measure temperature. In order to measure the heat flux passing through ($q_{meas}^{\prime \prime }$), half of the holes are placed on the upper side, and the other half is positioned exactly 20 mm ($Y_{t}$) under the upper ones. Thus, the aluminium block is partitioned into ten sections where the horizontal distance of the holes is equal. Thermal grease is applied to the holes before placing the thermocouples to ensure no air is trapped inside, which may affect the measurements. The arrangement is shown in Figure 2, where the base length ($B_{l}$) and width ($B_{w}$) are also indicated. A detailed discussion of heat flux measurements is given in Section 2.2.

A 20 mm thick plexiglass is placed on top of the module for visualization purposes and assembled with another plexiglass using M4 threaded rods. Thus, the top part of the aluminium block is pressed from both sides by these plexiglass parts. The aluminium block and the top plexiglass part had a special surface treatment to guarantee a perfect alignment and flatness to ensure no refrigerant passes between the channels. In addition to the surface treatment, the channel length of the module is kept at 150 mm, whereas a longer module will be more prone to refrigerant leakage among the channels. A 3D-printed o-ring is placed into the groove on the aluminium block to prevent leakage to the outside. At the inlet and outlet of the top plexiglass cover, a refrigerant pool is made for pressure measurements to check if the desired saturation pressure of R600a is reached. Placing pressure measurement holes at these pools eliminates the need for the calculation of pressure losses due to contraction or expansion if measurement had been taken from the piping network.

**Figure 3:**
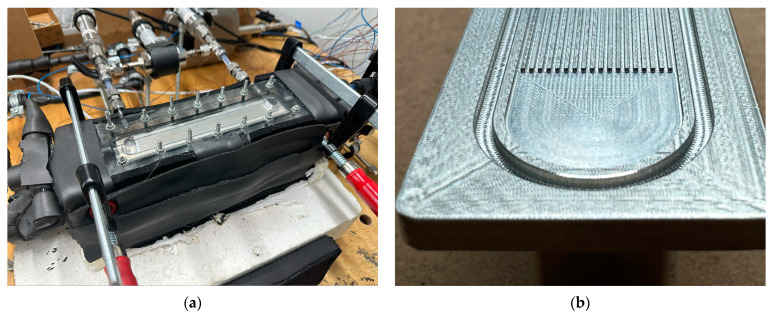
(**a**) Microchannel module assembled in the experimental rig; (**b**) a closer look at the aluminium block investigated.

The bare sides of the aluminium block assembly are first covered with silicone and then covered with thermal insulation sheets to ensure no heat loss occurs from the assembly to the ambient environment, which can be seen in Figure 3a. All tests are performed with an additional 20 mm thick heat insulation placed on top of the top plexiglass. The width of channels and location of thermocouple holes are measured with a Mitutoyo QuickScope optical measuring machine (made in Japan, uncertainty ±0.6 µm) and the height of channels with an LK/Nikon G90C GMM machine (made in the UK, uncertainty ±3.7 µm). A closer look at the investigated aluminium block can be seen in Figure 3b.

**Figure 4:**
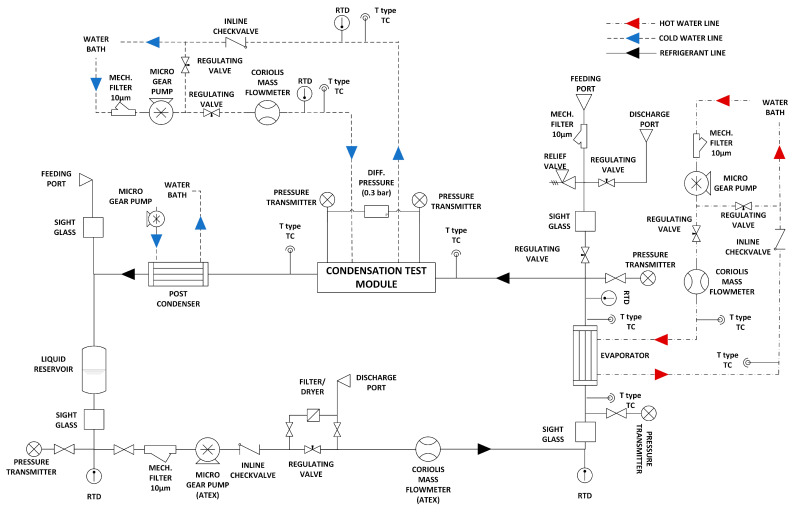
A schematic drawing of the experimental setup.

This aluminium block assembly is positioned in the refrigerant test loop where R600a is conditioned and circulated. The schematic of the experimental test setup is given in Figure 4. An ATEX-certified 24DC HNP Microsysteme mzr-7259Ex (made in Italy) micro gear pump is used for the circulation of R600a in the test rig. Micro gear pumps provide a smooth and steady mass flow without fluctuations; hence, they are used in two-phase microchannel flow studies. Before entering the micro gear pump, a particle filter is placed to protect the pump from contaminants. After the pump, a dryer-filter is placed to eliminate moisture or any other contaminants. Before each test starts, a cleaning process is made by circulating refrigerant through this line without conditioning. R600a then passes through an Endress–Hauser Promass A 300 Coriolis-type (made in Switzerland) mass flow meter for mass flow measurement.

After the mass flow meter, a shell and tube heat exchanger (evaporator) are placed to condition R600a to the desired vapour quality. In the inlet and outlet of the refrigerant line, Keller PR-33XEi-type (made in Switzerland) pressure transducers capable of measuring in the range of 0–10 bars and T-type thermocouples are placed for pressure and temperature measurements, respectively. A secondary heating loop is used to heat R600a with water supplied from a PolyScience AD15R-40-A12E-type (made in USA) water bath. This heating water is circulated by a Fluid-o-Tech MS (made in Italy) series micro gear pump to ensure a constant water supply. The mass flow rate of this heating water is measured by another Coriolis-type mass flow meter produced by the same manufacturer. Also, T-type thermocouples are placed on the inlet and outlet of this water line to measure the temperature of entering and leaving heating water. Conditioned R600a then leaves the evaporator and reaches the aluminium block assembly; another temperature measurement is logged in its vicinity to obtain an accurate inlet temperature of R600a.

Afterwards, R600a enters the aluminium block assembly and travels through microchannels. Conditioned coolant water passes through the bottom of the aluminium block to remove heat in order to perform condensation. Transferred heat from R600a to water is measured by 20 T-type thermocouples. This coolant loop also has a similar setup as the heating loop of the evaporator; T-type thermocouples are placed at the inlet and outlet of the water line to understand temperature change, a mass flowmeter is placed for mass flow measurement and coolant water is supplied from a separate water bath and circulated by a micro gear pump which all are the same brands as the heating loop.

**Figure 5:**
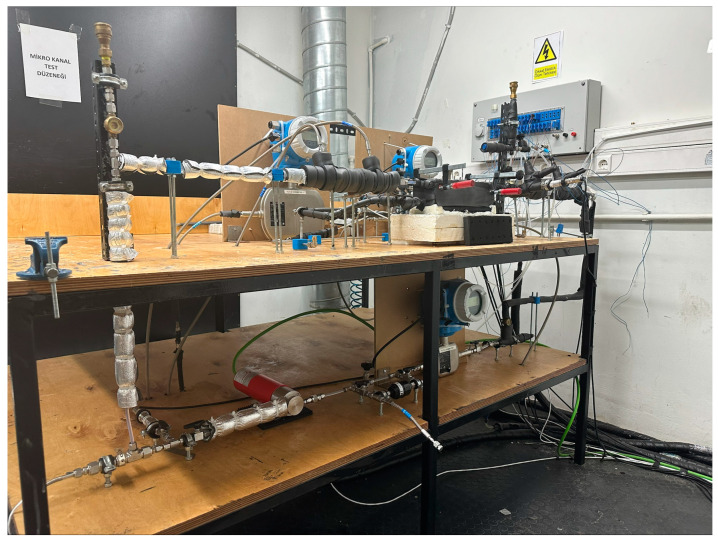
The refrigerant loop of the experimental test setup.

Leaving R600a enters the post condenser to obtain a fully condensed subcooled state with the help of a third water bath. This ensures no refrigerant reaches the R600a pump at the gas state to eliminate the risk of cavitation. This post condenser is also supplied with cooling water, which is circulated by another micro gear pump from a separate water bath. Finally, fully condensed R600a reaches the liquid reservoir and completes the loop. Additionally, several Class AA RTDs are mounted throughout the system to check whether measurements comply with each other and if any T-type thermocouple reading is not correct. The refrigerant loop can be seen in Figure 5.

The test setup is checked for leakages by applying nitrogen at 10 bars, and pressure change is tracked for at least 24 h. Before each test, the refrigerant loop is vacuumed by a vacuum pump and then charged with R600a from the charging port equipped with an inline filter placed at the highest point of the system. A needle valve is placed inside the loop to discharge the refrigerant. Also, a release valve pre-set to 11 bars is mounted to protect the test rig from an over-pressured condition.

**Figure 6:**
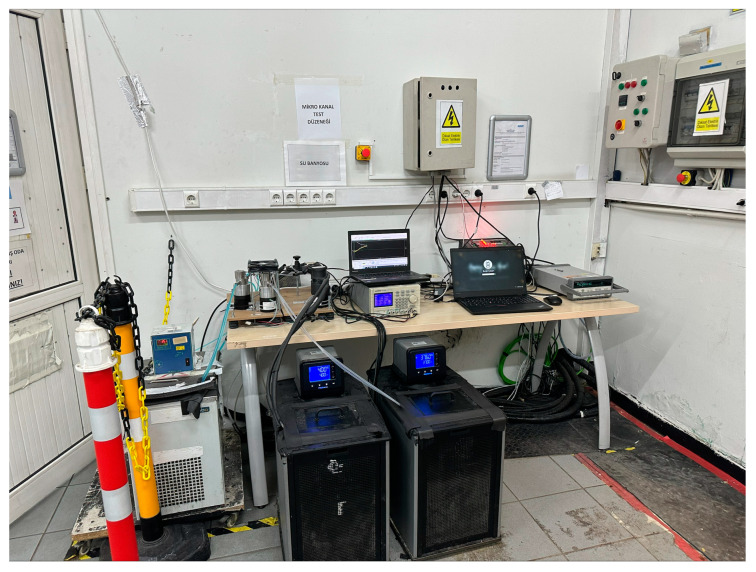
Secondary loop water baths, DAQ system, power supplies and electronic control units of the experimental setup.

Due to safety reasons, all equipment used in the refrigerant loop is ex-proof, and the use of electrical heaters or high-capacity reservoirs is avoided. All non-ex-proof system components, such as water baths of secondary heating and cooling loops, data acquisition equipment, power supplies and electronic control units, are placed outside of the test room, which can be seen in Figure 6.

### 2.2. Data Reduction

**Figure 7:**
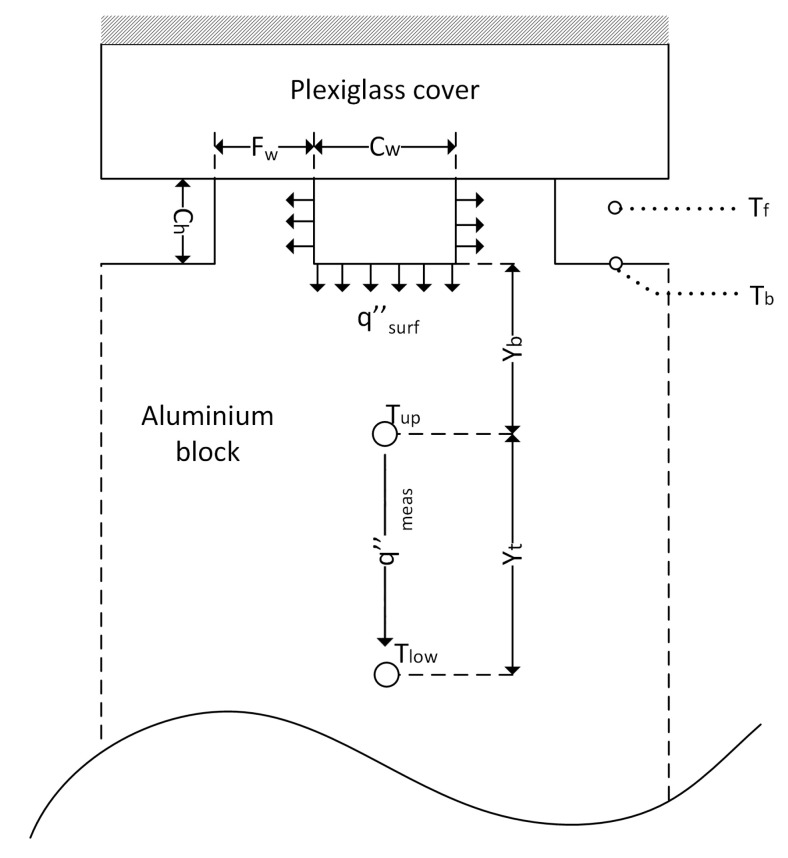
Microchannel aluminium block cross-section in detail (adapted from [6]).

As followed by [1,4,6], a fin analysis method is applied to understand the heat transfer rate from the refrigerant to the coolant, where the fin tip is assumed to be adiabatic, as shown in Figure 7. Conduction heat transfer through the aluminium block is assumed to be one-directional. The temperatures at upper $T_{up}$ and lower $T_{low}$ thermocouples are measured to find the heat flux passing through. The heat flux measured between these thermocouples ($q_{meas}^{\prime \prime }$) will be the same through the channel base and the upper thermocouple (distance denoted as $Y_{b}$) due to the 1D conduction assumption through the block. Afterwards, the base temperature of the channel ($T_{b}$) is found by using this heat flux value. Finally, using the known values of refrigerant and channel base temperatures, the condensation heat transfer coefficient (h) can be obtained due to the convection heat transfer from the refrigerant to the channel base. Dimensions and details of the aluminium block are given in Table 1 where $C_{h}$, $C_{w}$, $F_{w}$, N and $C_{L}$ indicate channel height, channel width, fin width, the number of channels and channel length, respectively.

Total heat transfer from refrigerant to the coolant, $q_{total}$ (W), and the heat flux measured between upper and lower thermocouples at the i^th^ section, $q_{meas,i}^{\prime \prime }$ (W/m^2^), can be written as follows:

(1) $$q_{total}=\sum_{i=1}^{10}A_{mb}q_{meas,i}^{\prime \prime }$$

(2) $$q_{meas,i}^{\prime \prime }=k_{Al}\frac{T_{up,i}-T_{low,i}}{Y_{t}}$$

where $k_{Al}$ is the thermal conductivity of the aluminium block (W/m·K), $A_{mb}=A_{b}/10$ denotes each section’s base area (m^2^) and $A_{b}$ is the total base area of the aluminium block.

Heat transfer through fins is accepted to be dissipated through the microchannel block body and becomes homogeneously distributed along the cross-section where the temperature measurement is taken. Thus, the local condensation heat transfer coefficient ($h_{i}$) can be calculated as follows:

(3) $$h_{i}=\frac{k_{Al}\left(T_{up,i}-T_{low,i}\right)\left(F_{w}+C_{w}\right)}{Y_{t}\theta_{b,i}\left(2\eta_{f,i}C_{h}+C_{w}\right)}$$

where $\theta_{b,i}=T_{f,i}-T_{b,I}$ is at the i^th^ section. For two-phase tests, the refrigerant temperature ($T_{f,i}$) at the i^th^ section is equal to the refrigerant saturation temperature $T_{f,sat,i}(P_{i})$.

Fin efficiency ($\eta_{f,i}$) and the fin parameter ($m_{i}$) at the i^th^ section are as follows:

(4) $$\eta_{f,i}=\frac{tanh\left(m_{i}C_{h}\right)}{m_{i}C_{h}}$$

(5) $$m_{i}=\sqrt{\frac{2h_{i}}{k_{Al}F_{w}}}$$

The channel base temperature ($T_{b,i}$) at the i^th^ section of the microchannel is calculated as follows:

(6) $$T_{b,i}=\frac{q_{meas,i}^{\prime \prime }Y_{b}}{k_{Al}}+T_{up,i}$$

The average heat transfer coefficient ($\overset{-}{h}$) can be written as follows:

(7) $$\overset{-}{h}=\frac{1}{81}\sum_{0.15}^{0.95}h(x)$$

where h(x) is calculated for each 0.01 interval of vapor qualities ranging from 0.15 to 0.95, as explained in Section 3.2.

The vapour quality ($x_{i}$) along the channel is calculated as follows:

(8) $$x_{i+1}=x_{i}-\frac{\left(q_{meas,i}^{\prime \prime }+q_{meas,i+1}^{\prime \prime }\right)A_{mb}}{2{\dot{m}}_{f}h_{fg}}$$

where ${\dot{m}}_{f}$ is the mass flow rate (kg/s), and $h_{fg}$ is the latent heat of vaporization (kJ/kg) of the refrigerant. If the refrigerant inlet vapour quality entering microchannels is desired to be lower than 1, then the inlet refrigerant vapour quality ($x_{in}$) is calculated as follows:

(9) $$x_{in}=\frac{{\dot{m}}_{hw}c_{p,hw}(T_{hw,in}-T_{hw,out})-{\dot{m}}_{f}c_{p,f}(T_{f,in,e}-T_{f,sat})}{{\dot{m}}_{f}h_{fg}}$$

where ${\dot{m}}_{hw}$, $c_{p,hw}$, $T_{hw,in}$ and $T_{hw,out}$ are the mass flow rate, the specific heat (kJ/kg·K) and the inlet and outlet temperatures of the heating water. $C_{p,f}$ and $T_{f,in,e}$ are the specific heat and the inlet temperature of the refrigerant entering the evaporator.

To check the energy balance between the sides for single-phase validation tests, the total heat transfer from the refrigerant ($q_{ref}$) to the coolant side ($q_{co}$) is calculated as follows, respectively:

(10) $$q_{ref}={\dot{m}}_{f}c_{p,f}\left(T_{f,in,m}-T_{f,out,m}\right)$$

(11) $$q_{co}={\dot{m}}_{co}c_{p,co}\left(T_{co,out}-T_{co,in}\right)$$

where ${\dot{m}}_{co}$, $c_{p,co}$, $T_{co,in}$ and $T_{co,out}$ are the mass flow rate, the specific heat (kJ/kg·K) and the inlet and outlet temperatures of the coolant water. $T_{f,in,m}$ and $T_{f,out,m}$ are the inlet and outlet temperatures of the refrigerant entering the microchannels.

The average cooling heat flux ($\bar{q_{co}^{\prime \prime }}$) is calculated as follows:

(12) $$\bar{q_{co}^{\prime \prime }}=\frac{\sum_{i=1}^{10}\left(q_{meas,\;i}^{\prime \prime }A_{mb}\right)}{A_{b}}$$

### 2.3. Operating Conditions and Experimental Uncertainty

The experimental uncertainty of the heat flux and the condensation heat transfer coefficient for each measurement is calculated as in [15] and shown in Table 2. The DAQ system to which T-type thermocouples are connected is calibrated by a FLUKE 5500A calibrator, and the uncertainty for thermocouples was measured as ±0.06 °C. Calibration reports and datasheets are used for the remaining measurement equipment.

For all tests performed in this study, the average uncertainties for heat fluxes and condensation heat transfer coefficients of each test are calculated in the range of 4.7–6.0% and 5.6–15.4%, respectively.

Tests are conducted for 35 °C, 40 °C and 45 °C saturation temperatures under 4 different mass fluxes (50, 66, 82 and 98 kg/m^2^·s) with varying inlet vapour quality (0.39–1.00). For all tests, the inlet coolant water temperature is kept at 10.3 °C with a mass flow rate of 11.6 g/s. The resulting average cooling heat flux applied was from 16.2 to 28.6 kW/m^2^. A summary of experimental operating conditions is given in Table 3, and the thermophysical properties of R600a are obtained from REFPROP v9.0 [16] and are shown in Table 4.

The highest mass flux investigated was 98 kg/m^2^·s, in which maximum saturation temperature change due to pressure loss through the inlet and outlet of the channels is measured to be less than 0.3 °C, which can be ignored. Higher mass fluxes than this limit would result in a higher channel pressure loss. This will cause a higher saturation temperature change in the refrigerant in the axial flow direction and will lead to inaccurate heat transfer coefficient measurements. Thus, higher mass fluxes can be investigated only when an accurate correction for saturation temperature due to pressure loss for each region and quality is present.

## 3. Results and Discussion

### 3.1. Single Phase Validation Tests

**Figure 8:**
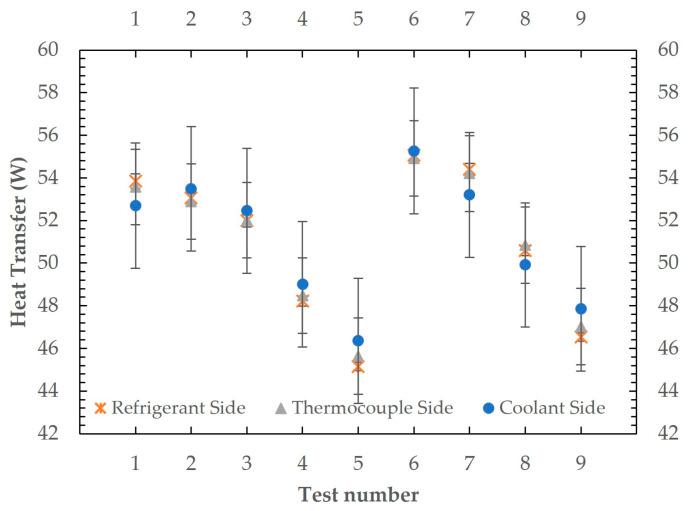
The measured total heat transfer rates from the coolant, the refrigerant and thermocouple sides in single-phase tests.

Before two-phase condensation tests, several single-phase tests with the R600a refrigerant are performed to validate the test rig’s measurement accuracy, as also performed by [14]. The test results and conditions can be seen in Figure 8 and Table 5, respectively.

In these tests, the coolant flow rate and inlet temperature were kept at 8.2 g/s and 5.2 °C where the refrigerant Re ranged from 955 to 1708. The refrigerant inlet temperature was 49 °C for tests 1–5 and was 51 °C for tests 6–9. The coolant flow rate was adjusted to obtain measurements within reasonable uncertainty limits. Measurements from the coolant side (Equation (11)), the refrigerant side (Equation (10)) and heat flux through thermocouples (Equation (1)) are taken to show their agreement. The maximum measurement difference between thermocouples and the refrigerant side (T-R) was 1.1% and the refrigerant side and the coolant side (R-C) was −2.8%. Measurement uncertainties for all tests are indicated in Table 5, and it can be seen that all measurement differences are in between uncertainty limits. These tests validate the energy balance between all measured sides along with the thermal conductivity of the aluminium block. The value of the thermal conductivity of the microchannel block is also validated by these tests, which is a better approach compared to using tabulated data or an analytical approach. During these tests, the maximum uncertainty of measurements from the refrigerant side and the thermocouples’ side was 3.9%; hence, this value is also considered as the uncertainty of the thermal conductivity of the aluminium block and stated in Table 2. The approach considers the effect of contact resistances and the actual thermal resistance of applied thermal paste, which may vary for every single application.

### 3.2. Two-Phase Condensation Tests

In total, 50 tests were conducted, and 452 data points were collected at 35 °C, 40 °C and 45 °C saturation temperatures with varying inlet vapour quality. When investigating high-latent heat refrigerants like R600a, a high amount of heat should be removed from the refrigerant if a full vapour quality range is desired to be covered at once. This raises the need for longer channels, which may lead to more challenging leakage control among channels due to increasing the risk of the plexiglass cover and microchannel module not being in absolute contact. Hence, this approach aims to investigate the possible effects of varying vapour quality on the heat transfer coefficient measurement.

**Figure 9:**
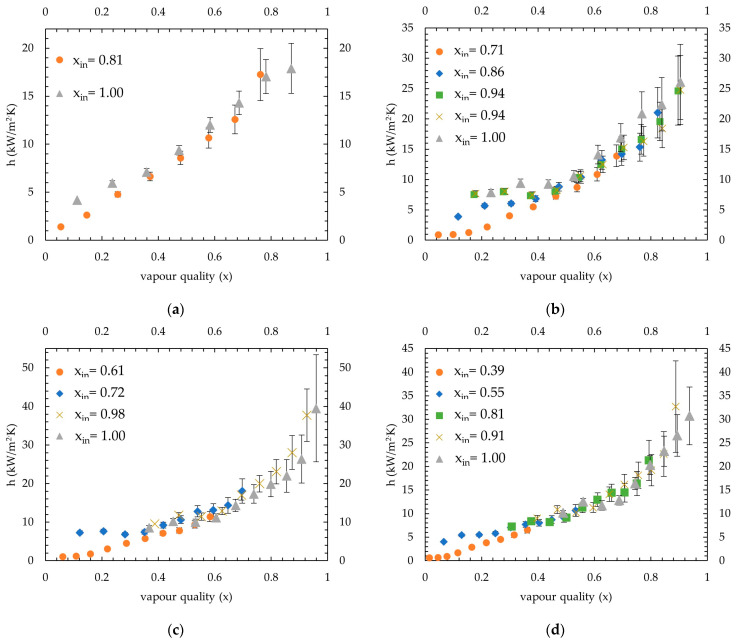
Condensation heat transfer coefficients at 35 °C saturation temperature: (**a**) at 50 kg/m^2^·s mass flux; (**b**) at 66 kg/m^2^·s mass flux; (**c**) at 82 kg/m^2^·s mass flux; and (**d**) at 98 kg/m^2^·s mass flux.

At 35 °C saturation temperature, the measured condensation heat transfer coefficients are shown in Figure 9. The applied average coolant heat fluxes ranged between 16.2 and 21.2 kW/m^2^, and the lowest inlet vapour quality introduced to the channels was 0.81, 0.71, 0.61 and 0.39 for 50 kg/m^2^·s, 66 kg/m^2^·s, 82 kg/m^2^·s and 98 kg/m^2^·s mass fluxes, respectively.

The measurements showed that the effect of varying inlet vapour quality seems to be more visible when a vapour quality lower than 0.3 is considered. When the refrigerant is introduced with a low vapour quality, a fully condensed state is reached within the channel length. It has been observed that this gives rise to a pressure fluctuation at the low vapour quality flow region, and fluid in this region starts to move forward and backward in the channels. The magnitude of fluctuation increased if the length of the subcooled state of the refrigerant became dominant compared to the two-phase region in the channels due to the velocity difference in each phase. The temperature of the subcooled liquid region decreases along the channel due to its single-phase nature, which also increases its density and lowers its velocity. This means that with the increasing length of the subcooled state, the temperature of the liquid phase decreases and, thus, its velocity. The gas phase encounters this liquid phase which would have lesser velocity as this length increases, which probably increases this fluctuation.

Hence, the same i^th^ section sees fully condensed liquid and low vapour quality two-phase refrigerant repeatedly. This results in measuring a lower heat transfer coefficient. The reason for this should be the lower heat transfer coefficient of the liquid phase, which decreases the average value measured. This effect can be clearly seen in Figure 9, the tests conducted for 35 °C saturation temperature for inlet vapour qualities 0.71 at 66 kg/m^2^s, 0.61 at 82 kg/m^2^s and 0.39 at 98 kg/m^2^s mass fluxes. The heat transfer coefficients measured for a vapour quality lower than 0.3 are much lower compared to the ones measured in higher inlet vapour quality tests. This is an expected outcome since the fluctuation affects this region; on the other hand, measurements of the near inlet region (higher than 0.3 vapour quality) are still aligned with higher inlet vapour quality experiments and seem to be less affected from the fluctuation.

Also, with increasing mass flux, the distance of the two-phase region in the channels becomes longer for the same inlet vapour quality. This created a lower fluctuating environment in the channels, which allowed for the investigation of much lower inlet vapour qualities. Thus, as can be seen in Figure 9, this measurement difference shifted through lower inlet vapour qualities from 0.71 to 0.39 for mass fluxes 66 kg/m^2^·s and 98 kg/m^2^·s, respectively.

At 50 kg/m^2^·s mass flux, the channel length was sufficient to observe from 0 to 1 vapour quality for all saturation temperatures; thus, there was no need to examine further lower inlet vapour qualities. For all 50 kg/m^2^·s mass flux cases, the length of the fluctuating part of the flow was long enough to prevent reaching a steady-state condition when an inlet vapour quality lower than 0.7 is introduced.

With increasing mass flux, the number of experiments is increased due to the needed long length to observe all vapour quality ranges. In addition, tests with the same inlet vapour quality are kept in the graphs to show the repeatability of experiments, especially at higher mass fluxes.

**Figure 10:**
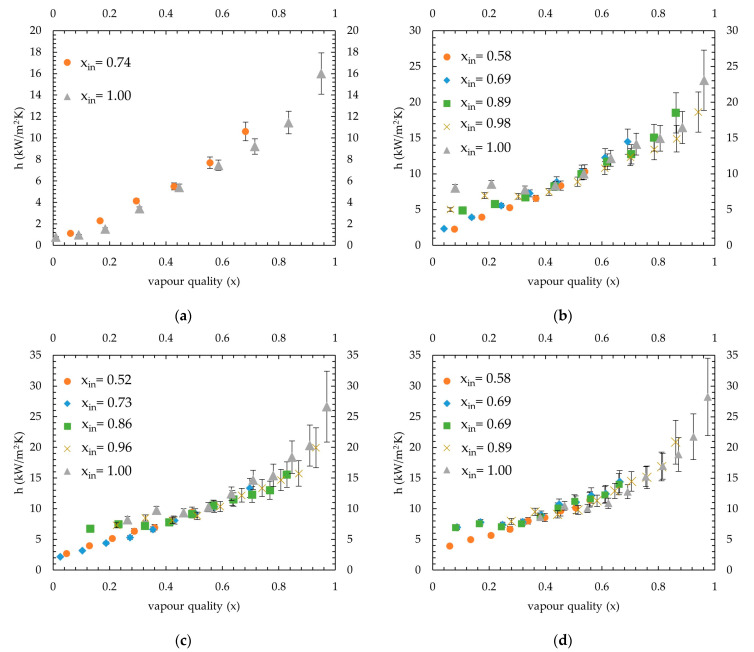
Condensation heat transfer coefficients at 40 °C saturation temperature: (**a**) at 50 kg/m^2^·s mass flux; (**b**) at 66 kg/m^2^·s mass flux; (**c**) at 82 kg/m^2^·s mass flux; and (**d**) at 98 kg/m^2^·s mass flux.

At 40 °C saturation temperature, the measured condensation heat transfer coefficients are shown in Figure 10. The applied average coolant heat fluxes ranged between 21.2 and 24.3 kW/m^2^, and the lowest inlet vapour quality introduced to the channels was 0.74, 0.58, 0.52 and 0.58 for 50 kg/m^2^·s, 66 kg/m^2^·s, 82 kg/m^2^·s and 98 kg/m^2^·s mass fluxes, respectively.

A similar outcome was also seen at 40 °C saturation temperature tests, for the lowest qualities such as 0.58, 0.52 and 0.58 introduced for 66 kg/m^2^·s, 82 kg/m^2^·s and 98 kg/m^2^·s mass fluxes, respectively. Also, the 0.73 inlet vapour quality showed a similar trend with 0.52 inlet vapour quality for 82 kg/m^2^·s mass flux. The effect of fluctuation can be well understood from the mass flux of the 66 kg/m^2^·s test, as shown in Figure 10b. As the inlet vapour quality decreased, a gradual decrease in the heat transfer coefficient was seen for the vapour qualities lower than 0.3. With decreasing inlet vapour quality, fluctuation becomes higher and affects the heat transfer coefficient measurement in this region highly. The local heat transfer coefficient at the 0.08 vapour quality was measured as 8 kW/m^2^·K and 2.3 kW/m^2^·K for inlet vapour quality tests with 1 and 0.58, respectively. At higher mass fluxes (82 kg/m^2^·s and 98 kg/m^2^·s), the gap between measurements decreased due to the decrease in fluctuation, as shown in Figure 10c,d. The same trend can also be seen in 35 °C saturation tests given in Figure 9c,d.

**Figure 11:**
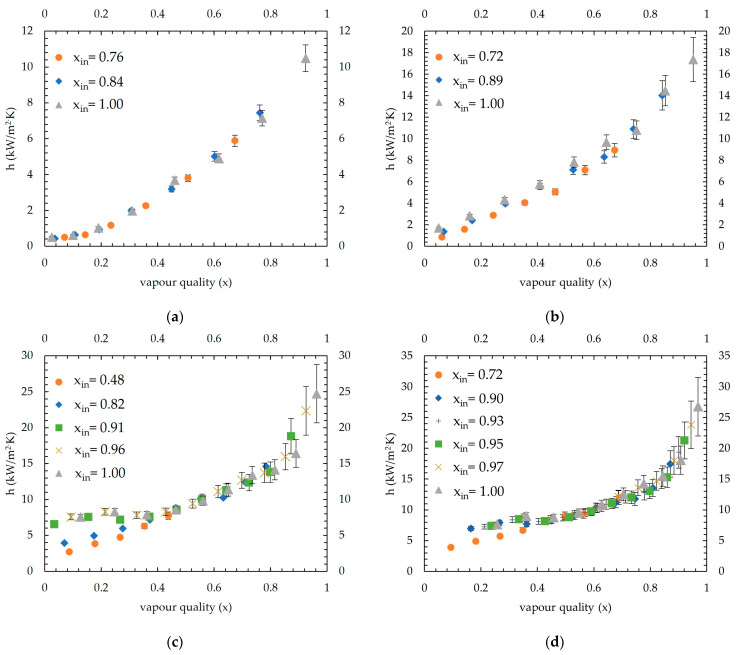
Condensation heat transfer coefficients at 45 °C saturation temperature: (**a**) at 50 kg/m^2^·s mass flux; (**b**) at 66 kg/m^2^·s mass flux; (**c**) at 82 kg/m^2^·s mass flux; and (**d**) at 98 kg/m^2^·s mass flux.

At 45 °C saturation temperature, the measured condensation heat transfer coefficients are shown in Figure 11. The applied average coolant heat fluxes ranged between 22.2 and 28.6 kW/m^2^, and the lowest inlet vapour quality introduced to the channels was 0.76, 0.72, 0.48 and 0.72 for 50 kg/m^2^·s, 66 kg/m^2^·s, 82 kg/m^2^·s and 98 kg/m^2^·s mass fluxes, respectively.

As seen in 35 °C and 40 °C tests, introducing low inlet vapour quality results in measuring a lower condensation heat transfer coefficient for 45 °C saturation measurements, too. Measurement differences can be seen at tests with 0.48 and 0.82 vapour quality inlets at 82 kg/m^2^·s mass flux and a 0.72 vapour quality inlet at 98 kg/m^2^·s mass flux as shown in Figure 11c,d. At 66 kg/m^2^·s mass flux, different from 35 °C and 40 °C saturation temperature tests, the plateau in the heat transfer coefficient seen for 0–0.4 vapour quality was not seen. This outcome can be attributed to higher cooling heat flux applied in the 45 °C condition, which probably changed the flow region as discussed in [1] and decreased the heat transfer coefficient for lower vapour qualities. This difference occurred at much lower quality inlets in 40 °C and 35 °C tests for the same mass fluxes. It can be understood that increasing cooling heat flux resulted in a shorter condensation length for the 45 °C condition, thus, much higher vapour quality inlets are affected by aforementioned fluctuation. Other than these low inlet quality tests, a good alignment among the tests for all mass fluxes can be seen, showing a good repeatability of the measurements.

Outcomes clearly suggest that, while performing varying inlet vapour quality tests, the pressure fluctuation of the flow should be low. It should be noted that this measurement difference is a result of a short length of two-phase flow, which may occur due to either applying high coolant heat flux or introducing refrigerant with low inlet vapour quality.

**Figure 12:**
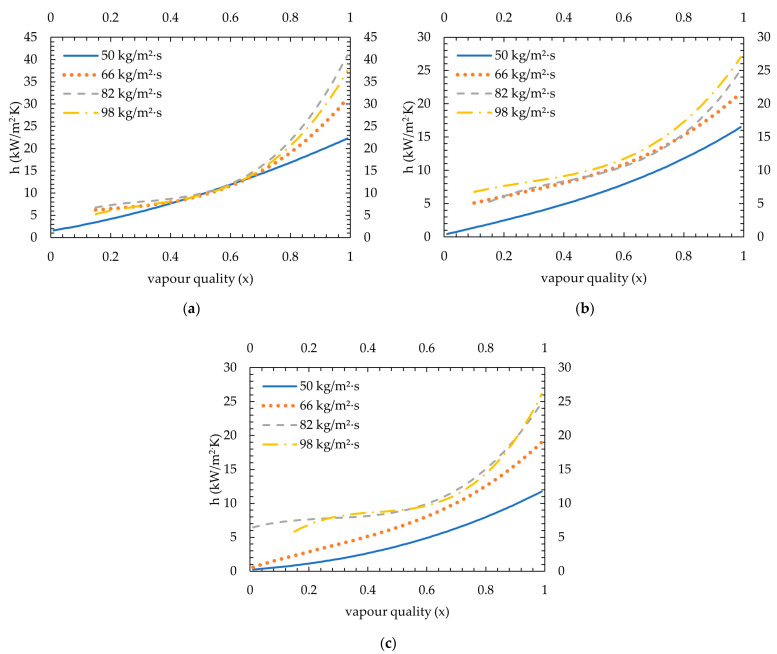
Condensation heat transfer coefficients for mass fluxes between 50 and 98 kg/m^2^·s: (**a**) at 35 °C saturation temperature; (**b**) at 40 °C saturation temperature; and (**c**) at 45 °C saturation temperature.

For all mass fluxes and saturation temperatures, given in Figure 9, Figure 10 and Figure 11, a third-order polynomial trendline is obtained for a clear comparison by removing low inlet vapour quality tests which show different trends. The excluded points were vapour qualities lower than 0.3 of the lowest vapour inlet quality tests of 66 kg/m^2^·s, 82 kg/m^2^·s and 98 kg/m^2^·s mass fluxes (average R^2^ found for 35 °C, 40 °C and 45 °C as 96%, 96% and 98%, respectively). The combined condensation heat transfer coefficients for all cases are shown in Figure 12. The average heat transfer coefficients for all four mass fluxes are calculated in the range of 0.15–0.95 vapour quality, considering that the highest of the lowest measured vapour quality remaining was 0.15 (see test 98 kg/m^2^·s mass flux at 45 °C saturation temperature in Figure 11d). Measurements show that the average heat transfer coefficients found for 35 °C were 11.3, 12.7, 14.4 and 13.4 kW/m^2^·K, for 50 kg/m^2^·s, 66 kg/m^2^·s, 82 kg/m^2^·s and 98 kg/m^2^·s, respectively. For 40 °C, it was measured as 7.6, 11, 11.3 and 12.6 kW/m^2^·K, respectively. At 45 °C saturation temperature, values measured were 4.6, 7.8, 11 and 11 kW/m^2^·K, respectively.

As can be seen in Figure 12, with decreasing saturation temperature, heat transfer coefficients increase as expected. At 50 kg/m^2^·s, a nearly 67% increase was measured from 45 °C to 40 °C saturation temperatures, whilst a further 48% increase was seen between 40 °C and 35 °C. This outcome is also stated in the work of [3] for the R1234ze(E) refrigerant, in which a gradual increase is shown from 70 °C to 30 °C saturation temperature. For R134a and R410A refrigerants, [4] reported an increase of 3–20% and 2–22% from 40 °C to 30 °C saturation temperatures, respectively. Rahman et al. [2] found that the heat transfer coefficient is higher at the 30 °C saturation temperature compared to 35 °C for R134a. As given in Table 4, the thermal conductivity of the liquid phase slightly increases with decreasing saturation pressure, which increases the rate of heat transfer from the vapour core to the channel base, as also concluded by [2,4]. Additionally, as stated in [2,14,17], interfacial shear stress plays an important role in the heat transfer coefficient. With decreasing saturation temperature, the density of the vapour decreases and the liquid increases. This results in a higher velocity difference between phases at the interphase, thus creating higher shear stress and increasing heat transfer between phases [2,14,17]. Also, the latent heat of vaporization ($h_{fg}$) increases with decreasing saturation temperature, which means more heat is released per mass unit flow.

Increasing mass flux increases heat transfer coefficients for all saturation temperatures up to a certain point. For 45 °C saturation temperature, increasing mass flux increases the heat transfer coefficient, whereas the difference between 82 kg/m^2^·s and 98 kg/m^2^·s nearly vanishes. A similar outcome can also be stated for 35 °C saturation temperature; even a slight decrease has occurred. Increasing the mass flux also increases the pressure drop due to increasing friction, which decreases the local saturation temperature and, therefore, the local fluid temperature. This effect diminishes the heat transfer coefficient difference above a specific mass flux. A similar outcome can be seen in the work of [6] for FC-72; heat transfer coefficients measured for 68 kg/m^2^·s and 118 kg/m^2^·s mass fluxes separated from 186, 248, 306 and 367 kg/m^2^·s mass fluxes, and the latter four have a similar heat transfer coefficient trend with changing vapour quality.

With decreasing saturation temperature, the difference in heat transfer coefficients between mass fluxes became insignificant, especially at lower vapour qualities. The measurements at 35 °C saturation temperature showed that heat transfer coefficients decreased with decreasing refrigerant mass flux, except 82 kg/m^2^·s had a slightly higher heat transfer coefficient than 98 kg/m^2^·s, between 1 and 0.6 but became similar onwards. It can be understood that until 0.6 vapour quality, the gas phase plays a dominant role and stays in contact with channel surfaces; thus, a higher gas phase Re results in a higher heat transfer coefficient. Also, when the liquid phase in the flow reaches a significant amount (lower than 0.6 vapour quality), a film layer is formed around channel surfaces, which increases thermal resistance, as mentioned before. Additionally, the temperature difference between the coolant and the refrigerant decreases from 45 °C to 35 °C, which reduces coolant mass flux and might play a role in reducing the effect of the refrigerant mass flux on heat transfer. A similar outcome can be seen in [2] for R134a at 35 °C saturation temperature for mass fluxes between 50 and 200 kg/m^2^·s; the heat transfer coefficients became nearly the same after 0.7 vapour quality. In the work of [3], for R134a, R236fa and R1234ze(E) refrigerants at 40 °C saturation temperature, a similar trend was shown for mass fluxes between 100 and 250 kg/m^2^·s.

Another expected outcome for the heat transfer coefficient is the decreasing trend with decreasing vapour quality, which is seen in all measurements. This outcome is a direct result of increasing the liquid amount in the channels.

### 3.3. Comparison to Correlations Stated in the Literature

A comparison of measurements is made to correlations stated in the literature, and the mean absolute errors (MAE) are calculated as follows:

(13) $$MAE=\frac{1}{E}\sum \frac{\left|h_{meas}-h_{corr,3}\right|}{h_{meas}}\times 100\%$$

where E is the number of data points.

Correlations given in the literature are predominantly stated for 4-sided heat transfer ($h_{corr,4}$); thus, every correlation is corrected to 3-sided heat transfer ($h_{corr,3}$) as the same procedure followed by [1,4,6,18]:

(14) $$h_{corr,3}=\left(\frac{{Nu}_{3}}{{Nu}_{4}}\right)h_{corr,4}$$

${Nu}_{3}$ and ${Nu}_{4}$ numbers are proposed by [19], and ${Nu}_{3}$ is correlated by [20], respectively:

(15) $${Nu}_{3}=8.235(1-1.833\beta +3.767\beta^{2}-5.814\beta^{3}+5.361\beta^{4}-2\beta^{5})$$

(16) $${Nu}_{4}=8.235(1-2.0421\beta +3.0853\beta^{2}-2.4765\beta^{3}+1.0578\beta^{4}-0.1861\beta^{5})$$

where β is the aspect ratio $C_{w}/C_{h}$.

**Figure 13:**
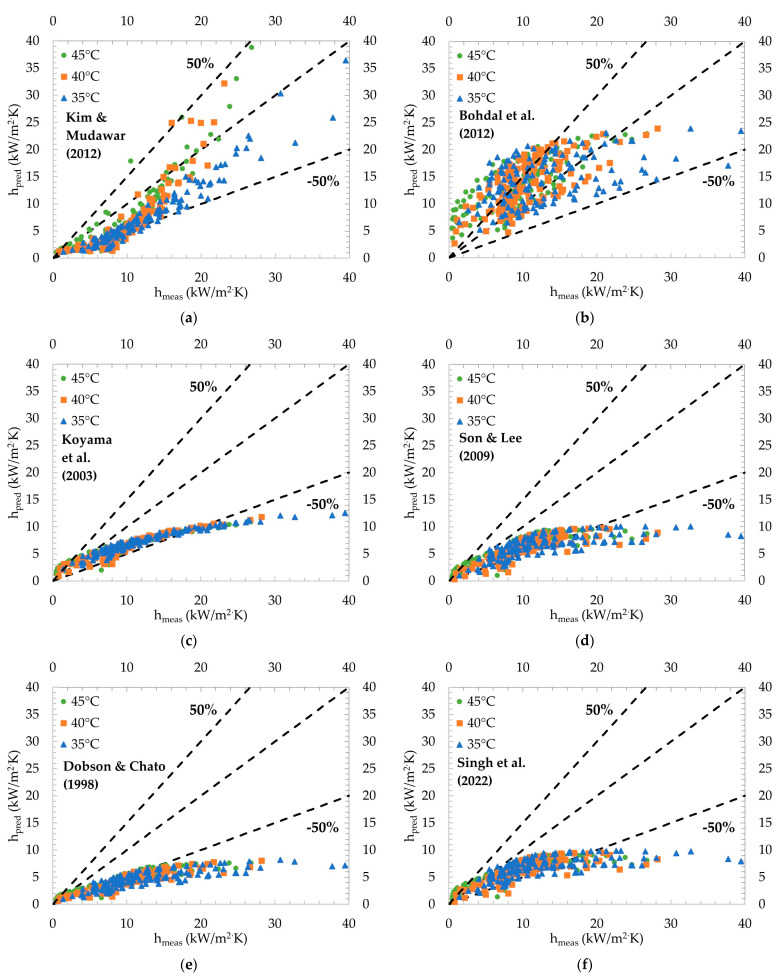
A comparison of the experimental results (h_meas_) to correlations (h_pred_) stated in the literature: (**a**) Kim and Mudawar [6]; (**b**) Bohdal et al. [5]; (**c**) Koyama et al. [23]; (**d**) Son and Lee [27]; (**e**) Dobson and Chato [21]; and (**f**) Singh et al. [4].

The MAEs of all correlations are shown separately for every saturation temperature, as in Table 6, and the most notable ones are given in Figure 13 for a better comparison. Correlations are given in Appendix A.

**Table 6:** The calculated MAE of correlations stated in the literature.

Reference	35 °C	40 °C	45 °C	All	# of Data in the ±50% Range
Singh et al. [4]	39%	38%	42%	40%	75%
Bohdal et al. [5]	53%	58%	127%	78%	63%
Kim & Mudawar [6]	42%	44%	39%	42%	67%
Dobson & Chato [21]	59%	56%	52%	56%	30%
Wang et al. [22]	61%	58%	52%	57%	18%
Koyama et al. [23]	36%	38%	49%	42%	85%
Huang et al. [24]	63%	62%	54%	60%	29%
Wang & Rose [25]	175%	208%	331%	237%	5%
Moser et al. [26]	67%	64%	64%	65%	10%
Son & Lee [27]	43%	41%	39%	41%	72%

The MAE was found as 40%, 41%, 42% and 42%, and data points that fell in the ±50% range are 75%, 72%, 67% and 85% for [4], [27], [6] and [23], respectively. None of these correlations were given for the R600a refrigerant and specifically mass fluxes studied in this work. Correlations given by [4,6] were obtained with a similar microchannel block assembly, where hydraulic diameters were 0.666–1 mm and 1 mm, respectively. Correlation given by Kim and Mudawar [6] predict heat transfer coefficients for higher vapour qualities much higher than measured, especially for 40 °C and 45 °C saturation temperatures. On the contrary, it underpredicts data measured for 35 °C saturation temperature. Moreover, a good prediction is obtained by the correlation given by [4], which slightly underpredicts heat transfer coefficients; the difference becomes wider for higher values. Similarly, predictions of [23,27] weaken for higher heat transfer values. As can be seen from Figure 9, Figure 10 and Figure 11, a steep increase occurs as vapour quality increases; thus, most correlations probably underpredict higher heat transfer coefficients for this reason. The correlation given by [5] predicts higher compared to the measurements of this study; however, with increasing vapour quality, the predictions become more aligned. Thus, the MAE calculated decreased from 45 °C to 35 °C because the measured heat transfer coefficients increased. Correlations by [21] and [26] are also given for macrochannels; as expected, they highly underpredicted the heat transfer coefficients, and only 30% and 10% fell in the ±50% range, respectively. Additionally, the correlation given by [24] was for an R410A–oil mixture in 1.6–4.18 mm diameter tubes; the MAEs found as 60% with 29% data were in the given range. The lowest accuracy was found for [25], where the MAE is 237%. This outcome is also found by [1,4], and as stated by [4], the probable reason is that the correlation is obtained theoretically rather than by measured data. Wang et al. [22] also showed poor agreement; even though the average MAE was 57%, only 18% of the data fell in the ±50% range.

As it can be seen from Table 6, no distinguishable difference between the saturation temperatures was observed; most correlations had a similar MAE for 35 °C, 40 °C and 45 °C except for [5,25]. The investigation of microchannels is too specific and given for a small range of parameters, making these correlations applicable to a narrow span, as also mentioned by [17]. Nearly all studies indicated higher MAEs when comparing their works to the literature and stating different correlations as acceptable.

## 4. Conclusions

The condensation heat transfer coefficients of R600a under 35 °C, 40 °C and 45 °C saturation temperatures in a multiport microchannel with a hydraulic diameter of 0.399 mm were investigated. Experiments were carried out for 50–98 kg/m^2^·s refrigerant mass fluxes with varying inlet vapour qualities. The findings are summarized below:

- The average heat transfer coefficients measured at 35 °C were 11.3, 12.7, 14.4 and 13.4 kW/m^2^·K; at 40 °C were 7.6, 11, 11.3 and 12.6 kW/m^2^·K; and at 45 °C were 4.6, 7.8, 11 and 11 kW/m^2^·K for mass fluxes ranging between 50 and 98 kg/m^2^s, respectively. These values consider data measured between 0.15 and 0.95 vapour qualities.
- Varying vapour quality does not significantly affect the heat transfer coefficients measured if the entering vapour quality allows for two-phase flow to be dominant in the channel. As the vapour inlet quality decreases, pressure fluctuations at the end region of the two-phase increase and deceive the measurement. This phenomenon becomes visible from a vapour quality lower than 0.3.
- Increasing mass flux increases the heat transfer coefficient up to a certain mass flux. Pressure loss due to friction lowers the saturation pressure of the fluid along with the saturation temperature. Thus, when investigating higher mass fluxes, the saturation pressure change should be taken into account.
- Decreasing saturation temperature increases the heat transfer coefficient due to changes in the thermophysical properties of R600a liquid and vapour.
- The heat transfer coefficient decreases with decreasing vapour quality due to the increasing liquid amount, which acts as a barrier between the gas core and the channel base.

## Figures and Tables

**Table 1:** Dimensions of the aluminium block investigated.

$C_{h}$ (mm)	$C_{w}$ (mm)	$D_{h}$ (mm)	$F_{w}$ (mm)	N (-)	$C_{L}$ (mm)	$B_{w}$ (mm)	$B_{l}$ (mm)	$Y_{t}$ (mm)	$Y_{b}$ (mm)
0.44	0.365	0.399	0.435	19	150	16.8	150	20	9.6

**Table 2:** The measurement uncertainties of the equipment used and calculated uncertainties for parameters investigated.

Equipment/Parameter	Uncertainty
T-type thermocouples	±0.06 °C
Coriolis mass flow meters	0.1% of the reading
Pressure transducers	±1.6 mbar
The thermal conductivity of the aluminium block (W/m·K)	3.9%
RTDs (4 wire, Class AA)	±(0.1 + 0.0017 × |T|) °C
$\mathrm{Heat}\;\mathrm{flux}\;(q_{meas}^{\prime \prime }$)	4.7–6.0%
Condensation heat transfer coefficient (h)	5.6–15.4%

**Table 3:** Experimental operating conditions of this study.

Refrigerant	R600a
Saturation pressures (bar)	4.65–6.04
Saturation temperatures (°C)	35, 40 and 45
Refrigerant mass flux (kg/m^2^·s)	50, 66, 82 and 98
Coolant	water
Coolant mass flow rate (g/s)	11.6
Coolant inlet temperature (°C)	10.3
Vapour inlet quality	0.39–1.00
Average cooling heat flux (kW/m^2^)	16.2–28.6

**Table 4:** Thermophysical properties of R600a [16].

		Saturation Temperature		
Equipment	Unit	35 °C	40 °C	45 °C
Pressure	(bar)	4.6477	5.3121	6.0445
Liquid Density	(kg/m^3^)	537.83	531.19	524.37
Vapor Density	(kg/m^3^)	11.988	13.666	15.529
$\mathrm{Liquid}\;c_{p}$	(kJ/kg·K)	2.4982	2.5349	2.5737
$\mathrm{Vapor}\;c_{p}$	(kJ/kg·K)	1.8771	1.921	1.9675
Heat of Vaporization	(kJ/kg)	317.53	311.52	305.27
Liquid thermal conductivity	(W/m·K) × 10^−3^	85.752	84.051	82.387
Vapour thermal conductivity	(W/m·K) × 10^−3^	17.937	18.524	19.136
Liquid viscosity	(µPa-s)	136.2	129.39	122.96
Vapour viscosity	(µPa-s)	7.7693	7.9125	8.0616
Surface Tension	(N/m) × 10^−3^	8.9689	8.4105	7.8585

**Table 5:** Single-phase validation test conditions and measured data.

		Total Heat Transfer (W)			Difference (%)		Uncertainty (%)		
Test #	Re	Refrigerant Side (R)	Thermocouples Side (T)	Coolant Side (C)	T-R	R-C	R	T	C
1	1708	53.8	53.6	52.7	−0.5	2.1	0.6	3.3	5.3
2	1580	53.1	52.9	53.5	−0.3	−0.8	0.6	3.4	5.2
3	1456	52.0	52.0	52.5	0.1	−0.9	0.6	3.4	5.3
4	1207	48.2	48.5	49.0	0.6	−1.6	0.5	3.7	5.7
5	955	45.1	45.6	46.4	1.1	−2.7	0.4	3.9	6.0
6	1599	55.1	54.9	55.3	−0.3	−0.4	0.6	3.2	5.1
7	1453	54.4	54.2	53.2	−0.3	2.2	0.5	3.3	5.3
8	1206	50.6	50.8	49.9	0.5	1.3	0.5	3.5	5.6
9	955	46.5	47.0	47.9	1.0	−2.8	0.4	3.8	5.8

## Data Availability

The original contributions presented in the study are included in the article, further inquiries can be directed to the corresponding authors.

## Appendix A

The heat transfer coefficient $h_{corr,4}$ can be calculated by the following:

(A1) $$h_{corr,4}=\frac{Nuk_{l}}{D_{h}}$$

for correlations given as Nu, where $k_{l}$ is the thermal conductivity (W/m·K) of the saturated liquid refrigerant.

**Reference**	**Correlation**	**Conditions**
Singh et al. [4]	$Nu=0.0815{Re}_{l}^{0.73}{Pr}_{l}^{0.93}{\left(\frac{1}{\beta }\right)}^{0.05}{\left(\frac{1}{X_{tt}}\right)}^{0.68}$	$D_{h}$: 0.666–1 mm, Fluids: R134a, R410A, mass fluxes: 200–600 kg/m^2^·s, $T_{f,sat}$: 30–40 °C, x: 0.05–0.83, multi-channel
Bohdal et al. [5]	$Nu=25.084{Re}_{l}^{0.258}{Pr}_{l}^{-0.495}p_{r}^{-0.288}{\left(\frac{x}{1-x}\right)}^{0.266}$	$D_{h}$: 0.31–3.3 mm Fluids: R134a, R404A, R407C, mass fluxes: 100–1300 kg/m^2^·s, $T_{f,sat}$: 20–50 °C, x: 0–1, single-channel
Kim and Mudawar [6]	$h_{corr,3}=\left(\frac{{Nu}_{3}}{{Nu}_{4}}\right)\left(0.1+0.06{Pr}_{l}^{0.8}\right){Pr}_{l}^{-1}{Re}_{l}^{-0.13}c_{p,l}$ $\times \sqrt{\frac{fG^{2}{\left(1-x\right)}^{2}}{2}\left(1+\frac{21\left[1-exp(-0.319D_{h})\right]}{X}+\frac{1}{X^{2}}\right)}$ See [6] for f Fanning friction factor.	Given for 3-sided condensation, $D_{h}$: 1mm, Fluid: FC-72, mass fluxes: 68–367 kg/m^2^·s, $T_{f,sat}$: 57.2–62.3 °C, multi-channel
Dobson and Chato [21]	$Nu=0.023{Re}_{l}^{0.8}{Pr}_{l}^{0.4}\left(1+\frac{2.22}{X_{tt}^{0.89}}\right)$	$D_{h}$: 3.14–7.04 mm, Fluids: R134a, R12, R22, R32/R125
Wang et al. [22]	$Nu=0.0274{Pr}_{l}{Re}_{l}^{0.6792}x^{0.2208}{\left(\frac{1.376+8X_{tt}^{1.655}}{X_{tt}^{2}}\right)}^{0.5}$	$D_{h}$: 1.46, Fluid: R134a, mass fluxes: 75–750 kg/m^2^·s, $T_{f,sat}$: 61.5–66 °C multi-channel
Koyama et al. [23]	$Nu=\sqrt{{Nu}_{F}^{2}+{Nu}_{B}^{2}}$ ${Nu}_{F}=0.0152\left(1+0.6{Pr}_{l}^{0.8}\right)\left(\frac{\emptyset_{v}}{X_{tt}}\right){Re}_{l}^{0.77}$ $\emptyset_{v}^{2}=1+21\left(1-e^{-0.319D_{h}}\right)X_{tt}+X_{tt}^{2}$ ${Nu}_{B}=0.725H\left(\xi \right){\left(\frac{Ga{Pr}_{l}}{Ph}\right)}^{0.25}$ $H(\xi )=\xi +\left\{10\left[{\left(1-\xi \right)}^{0.1}-1\right]+1.7\left(10^{-4}\right){Re}_{lo}\right\}\sqrt{\xi }\left(1-\sqrt{\xi }\right)$ $Ga=\frac{g\rho_{l}^{2}D_{h}^{3}}{\mu_{l}^{2}}$ $Ph=\frac{c_{p,l}\left(T_{f,sat}-T_{b}\right)}{h_{fg}}$ $\xi ={\left[1+\frac{\rho_{v}}{\rho_{l}}\left(\frac{1-x}{x}\right)\left(0.4+0.6\sqrt{\frac{\frac{\rho_{l}}{\rho_{v}}+0.4\frac{1-x}{x}}{1+0.4\frac{1-x}{x}}}\right)\right]}^{-1}$	$D_{h}$: 0.8–1.11 mm, Fluid: R134a, mass fluxes: 100–700 kg/m^2^·s, $T_{f,sat}$: 60.5 °C, multi-channel
Huang et al. [24]	$Nu=0.0152(-0.33+0.83{Pr}_{l}^{0.8})\frac{\phi_{v}}{X_{tt}}{Re}_{l}^{0.77}$ $\phi_{v}=1+0.5{\left[\frac{G}{{\left(gD_{h}\rho_{v}\left(\rho_{l}-\rho_{v}\right)\right)}^{0.5}}\right]}^{0.75}X_{tt}^{0.35}$	$D_{h}$: 1.6–4.18 mm, Fluids: R410A-oil, mass fluxes: 200–600 kg/m^2^·s, x: 0.3–0.9 $q_{co}$: 4.23–19.07 kW/m^2^
Wang and Rose [25]	$Nu=1.41\left(\frac{\rho h_{fg}\sigma b}{\mu_{l}k_{l}\Delta T}\right)$ $\Delta T=T_{f,sat}-T_{b}$	1–2 mm side length square, 1 mm side length triangle, 1.5 × 1 rectangle, Fluids: R134a, R22, R410A, Ammonia, R152a, R290, CO_2_, $T_{f,sat}$: 25–50 °C
Moser et al. [26]	$Nu=\frac{0.0994^{C_{1}}{Re}_{l}^{C_{2}}{Re}_{eq}^{1+0.875C_{1}}{Pr}_{l}^{0.815}}{(1.58\ln{Re}_{eq}-3.28)(2.58\ln{Re}_{eq}+13.7{Pr}_{l}^{2/3}-19.1)}$ $C_{1}=0.126{Pr}_{l}^{-0.448}$ $C_{2}=-0.113{Pr}_{l}^{-0.563}$ ${Re}_{eq}=\phi_{lo}^{8/7}{Re}_{lo}$ $\phi_{lo}^{2}=A_{1}+\frac{3.24A_{2}}{{Fr}^{0.045}{We}^{0.035}}$ $A_{1}={\left(1-x\right)}^{2}+x^{2}\left(\frac{\rho_{l}}{\rho_{v}}\right)\left(\frac{f_{\mathrm{vo}}}{f_{lo}}\right)$ $A_{2}=x^{0.78}{\left(1-x\right)}^{0.24}{\left(\frac{\rho_{l}}{\rho_{v}}\right)}^{0.91}{\left(\frac{\mu_{v}}{\mu_{l}}\right)}^{0.19}{\left(1-\frac{\mu_{v}}{\mu_{l}}\right)}^{0.7}$ $Fr=\frac{G^{2}}{gD_{h}\rho_{tp}^{2}}$ $We=\frac{G^{2}D}{\rho_{tp}\sigma }$ $\rho_{tp}={\left(\frac{x}{\rho_{v}}+\frac{1-x}{\rho_{l}}\right)}^{-1}$	$D_{h}$: 3.14–20 mm, Fluids: R11, R12, R22, R125, R134a, R410A, mass fluxes: 87–862 kg/m^2^·s
Son and Lee [27]	$Nu=0.034{Re}_{l}^{0.8}{Pr}_{l}^{0.3}\left[3.28{\left(\frac{1}{X_{tt}}\right)}^{0.78}\right]$	$D_{h}$: 1.5–6 mm, Fluids: R22, R134a, R410A, mass fluxes: 200–400 kg/m^2^·s, x: 0.05–0.95, $q_{co}$: 5–35 kW/m^2^, ${Re}_{l}$:500–10,000, ${Pr}_{l}$: 2–5, $\frac{1}{X_{tt}}$: 1–40, single-channel
	where β is aspect ratio ($C_{w}/C_{h}$), $X_{tt}$ is turbulent–turbulent Martinelli parameter, X is Martinelli parameter, ${Re}_{l}$ is liquid Reynolds number, ${Pr}_{l}$ is liquid Prandtl number, $p_{r}$ is reduced pressure, G is mass flux (kg/m^2^·s), ${Re}_{lo}$ is liquid-only Reynolds number, Ga is Galileo number, g is gravitational acceleration (m/s^2^), Fr is Froude number, We is Weber number, b is length of side of channel (mm), $\rho_{v}$ is density of refrigerant vapour (kg/m^3^), $\rho_{l}$ is density of liquid refrigerant (kg/m^3^), ${Re}_{eq}$ is equivalent Reynolds number, and $f_{vo}$ and $f_{lo}$ are vapour-only and liquid-only friction factors.	
